# Acceptance and commitment therapy for people with acquired brain injury: Rationale and description of the BrainACT treatment

**DOI:** 10.1177/02692155231154124

**Published:** 2023-02-07

**Authors:** Johanne CC Rauwenhoff, Yvonne Bol, Caroline M van Heugten, Tim Batink, Chantal AV Geusgens, Anja JHC van den Hout, Peter Smits, Christianne RT Verwegen, Annemarie Visser, Frenk Peeters

**Affiliations:** 1 School for Mental Health and Neuroscience, Faculty of Health, Medicine and Life Sciences, 5211 Maastricht University, Maastricht, The Netherlands; 2 Limburg Brain Injury Centre, Maastricht, The Netherlands; 3 Department of Clinical and Medical Psychology, Zuyderland Medical Centre, Sittard-Geleen/Heerlen, The Netherlands; 4 Department of Neuropsychology and Psychopharmacology, Faculty of Psychology and Neuroscience, 5211 Maastricht University, Maastricht, The Netherlands; 5 Faculty of Psychology and Educational Sciences, Open University, Heerlen, The Netherlands; 6 Department of Medical Psychology, MUMC+, Maastricht, The Netherlands; 7 Department of Rehabilitation Medicine, Sint Maartenskliniek, Nijmegen, The Netherlands; 8 Department of Rehabilitation Medicine, Center for Rehabilitation, University Medical Centre Groningen, Haren, The Netherlands; 9 Department of Clinical Psychological Science, Faculty of Psychology and Neuroscience, 5211 Maastricht University, Maastricht, The Netherlands

**Keywords:** Acceptance and commitment therapy, acquired brain injury, anxiety, depression

## Abstract

**Background:**

The treatment of anxiety and depressive symptoms following acquired brain injury is complex and more evidence-based treatment options are needed. We are currently evaluating the BrainACT intervention; acceptance and commitment therapy for people with acquired brain injury.

**Rationale:**

This paper describes the theoretical underpinning, the development and content of BrainACT. Acceptance and commitment therapy focuses on the acceptance of feelings, thoughts and bodily sensations and on living a valued life, without fighting against what is lost. Since the thoughts that people with acquired brain injury can experience are often realistic or appropriate given their situation, this may be a suitable approach.

**Theory into practice:**

Existing evidence-based protocols were adapted for the needs and potential cognitive deficits after brain injury. General alterations are the use of visual materials, summaries and repetition. Acceptance and commitment therapy-specific adaptions include the Bus of Life metaphor as a recurrent exercise, shorter mindfulness exercises, simplified explanations, a focus on experiential exercises and the monitoring of committed actions. The intervention consists of eight one-hour sessions with a psychologist, experienced in acceptance and commitment therapy and in working with people with acquired brain injury. The order of the sessions, metaphors and exercises can be tailored to the needs of the patients.

**Discussion:**

Currently, the effectiveness and feasibility of the intervention is evaluated in a randomised controlled trial. The BrainACT intervention is expected to be a feasible and effective intervention for people with anxiety or depressive symptoms following acquired brain injury.

## Introduction

Acquired brain injury can lead to physical disabilities, persistent cognitive deficits, behavioural dysregulations and emotional consequences.^1^ People with acquired brain injury are at an increased risk of developing symptoms of depression and anxiety.^2^ These symptoms are related to decreased participation (dependency in daily life and lower return-to-work rates), higher re-hospitalisation rates and increased cognitive and physical impairments.^3^ The treatment of acquired brain injury-related anxiety and depressive symptoms is challenging. The evidence for the effectiveness of psychotherapies for depression symptoms is less robust for patients with brain injury than for depressed patients without brain injury. Reviews found that psychotherapies for mood symptoms following acquired brain injury either have small short-term effects or the effects did not differ from control conditions.^4–8^ More effective evidence-based treatment options are necessary for treating acquired brain injury-related anxiety and depressive symptoms.

Previous articles have pointed out the possible beneficial effect of acceptance and commitment therapy for people with acquired brain injury.^9,10^ Furthermore, several studies showed that acceptance and commitment therapy can be effective for people with traumatic brain injury^11,12^ and stroke.^13^ However, more studies are needed to investigate the short and long-term effects of acceptance and commitment therapy specifically adapted for brain injury-related anxiety and depressive symptoms. Furthermore, large-scale group studies are needed to examine the effectiveness of acceptance and commitment therapy for stroke survivors.

The possible cognitive deficits of people with acquired brain injury can form a challenge during therapy.^14^ When treating patients with acquired brain injury, it is essential to combine knowledge of psychotherapeutic processes and neuropsychological consequences of the acquired brain injury (i.e., neuropsychotherapy) and adapt psychotherapy accordingly.^15^

Recently, we developed the BrainACT intervention. This is an acceptance and commitment therapy intervention modified to the needs and possible cognitive deficits of people with acquired brain injury. The effectiveness of the BrainACT treatment was studied using a single case experimental design, where three out of the four participants experienced improvements regarding, anxiety and depressive symptoms, cognitive fusion, quality of life and stress.^16^ Currently, the BrainACT intervention is further being evaluated in a multicentre randomised controlled trial.^17^ In this article, we present the rationale and description of the BrainACT intervention using the Template for Intervention Description and Replication framework and illustrate it with a case description.

## The Rationale, Theory and Goal of Acceptance and Commitment Therapy

Acceptance and commitment therapy is a third-wave behavioural therapy with roots in the Relational Frame Theory. According to Relational Frame Theory, human suffering predominantly emerges from normal psychological processes, especially those involving human language.^18^ Acceptance and commitment therapy tries to decrease the influence of the verbal content of cognition that can cause avoidant behaviour and dismantle ineffective rule-governed behaviours.^19^ As acceptance and commitment therapy assumes that (psychological) pain is inherent to being human and in itself does not lead to the development of psychopathology, the main aim of acceptance and commitment therapy is not to reduce symptoms, but to promote well-being.^18,20^ The stress or negative thoughts that people with acquired brain injury can experience are often realistic or appropriate given their situation.

An approach that focuses on acceptance might be more beneficial than an approach focusing on eliminating and altering thoughts and feelings related to acquired brain injury.^21^ Acceptance and commitment therapy focuses on the acceptance of feelings, thoughts and bodily sensations and on living a valued life, without fighting against what is lost This would indicate an increase in psychological flexibility, which is the main treatment goal of acceptance and commitment therapy and is described as “the ability to contact the present moment more fully as a conscious human being and to change, or persist in, behaviour when doing so serves valued ends.”^22^

Psychological flexibility is established through the six core components of acceptance and commitment therapy: acceptance (accepting feelings without changing them), cognitive defusion (creating a distance from thoughts), self-as-context (taking perspective and separating the self from the process of thinking), mindfulness (attention for the here and now), values (discovering what really matters) and committed action (actions linked to chosen values). These core processes both overlap and interact with each other.^22^

To rely less heavily on language, each of the six core components is demonstrated through active experiential practice, such as experiential exercises (mindfulness and defusion exercises), behavioural change processes (committed action) and the use and acting out of metaphors.^19^ As a result, the abstract becomes more concrete and implicit memory resources are engaged to support learning.^23^ The therapist actively models the acceptance and commitment therapy processes and participates together with the patient in the exercises as an equal.^24^ Metaphors in acceptance and commitment therapy are used to validate the patient's experience, to let the patient understand and become aware of their situation and to see their situation from another perspective. A metaphor sidesteps analytical language and uses a more experiential way of learning.^18^ In addition, when comparing their situation with a metaphor, patients step out of their language system and are therefore less susceptible to cognitive fusion.^25^ Since the acceptance and commitment therapy community strives towards an open-source ethos, there are pictures and videos available of metaphors that can be used during therapy. This can be useful since visual resources can aid metaphor comprehension.

## What is BrainACT: Methods and Protocol Development

### Adapting Psychotherapy and Acceptance and Commitment Therapy for People with Acquired Brain Injury

Based on a literature review, several alterations of psychotherapy were identified: memory aids (visual resources, workbook, session summaries, written notes), simplified tasks, emphasis on behavioural techniques, repetition of content, structured sessions, incorporating breaks, use of concrete examples, involving relatives in therapy or homework tasks, one-on-one face-to-face sessions, adjusting the number of sessions and the duration of the sessions, and including an initial session focused on psycho-education and normalising reactions to acquired brain injury.^9,26–28^ To consolidate acceptance and commitment therapy skills in daily life, Kangas and McDonald^9^ recommended that sessions should be on a weekly basis in the initial phase and thereafter less frequent.

Modifications specific to acceptance and commitment therapy can include: summaries of the core components covered in each session, focussing on experiential exercises which are audiotaped, using concrete and personally relevant metaphors and exercises, using shorter mindfulness exercises, providing tangible ideas (for instance the card sorting task as an aid for value exploration), and focussing on promoting values-based goal-directed behaviour and behavioural activation components.^9,10,27^ Furthermore, when providing acceptance and commitment therapy to people with acquired brain injury, it is warranted to use one metaphor at a time and to frequently repeat this metaphor. Furthermore, therapists should not adhere too much to the original meaning of the metaphor but should allow patients to develop their own meaning.^27^ Finally, patients should be able to reflect on how the brain injury has prevented them from engaging in valuable activities and how to engage in valuable activities with physical and/or cognitive limitations.^9^

### Development of the Intervention

The findings from the literature review described above were the starting point of the development process, which is shown in Table 1. These alterations were presented to an expert group of five psychologists (Authors TB, CG, AvdH, PS, and CV), experienced in working with patients with acquired brain injury and/or experienced with acceptance and commitment therapy. Furthermore, the psychologists were employed in different healthcare facilities. A brainstorm session took place to discuss the layout and focus of the intervention, which metaphors and exercises should be included in the intervention, and how to shape the workbook of the patients. Based on input from these discussions, the literature review and existing interventions^11,29,30^ the initial version of the protocol was created. The expert group was requested to provide feedback on the initial version, and based on their feedback, the first version of the protocol was developed.

**Table 1.** Adapting acceptance and commitment therapy for patients with acquired brain injury.

**Adaptations based on literature review and expert group meetings**	
General adaptations: - Individual - Structure and repetition - Concrete material - Summaries of the sessions (visual and auditory) - Weekly sessions at the start, then more spread out	Acceptance and commitment therapy-specific adjustments: - One concrete and relevant metaphor to explain multiple core components - Visualisation of metaphors - Value based behaviour is followed up in every session - Short mindfulness exercises
**First concept of the treatment protocol**	
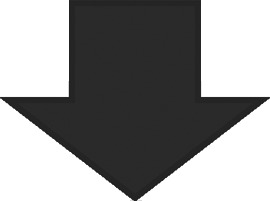	
Pilot study	
Four patients with acquired brain injury and three psychologists gave feedback on the protocol	
Major changes afterwards: - 60-min sessions instead of 90 min - Decreased content of the sessions - Simplification of metaphors - Adding ‘translations**’** of the metaphors to the situation of the patient - Simplified the session on self-as-context - Flexible order of the sessions	
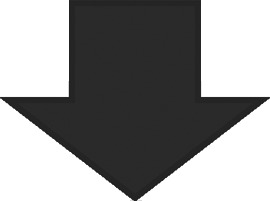	
**Version of the protocol currently evaluated for effectiveness and presented in this article**	

This version was tested in a single case experimental design study on four patients with acquired brain injury.^16^ After completing the treatment, all participants and psychologists were asked for feedback on the protocol via a semi-structured interview. Patients and psychologists were interviewed about the length, order, frequency of sessions, the metaphors, the (homework) exercises and the materials used in the sessions. Patients were asked to rate the intervention, whether they benefited from the treatment and whether they would recommend the treatment to other patients. Psychologists were asked whether they thought the patient benefited from the treatment and whether it was sufficiently adapted for the possible cognitive deficits and needs of people with acquired brain injury. Based on their feedback and suggestions a final version of the protocol was developed. This version is now being evaluated in a multicentre randomised controlled trial.

## Procedures: How, What, Where, and for Whom?

### Target Group and Setting

The intervention is developed for patients who experience depression and/or anxiety symptoms following acquired brain injury regardless of time since injury. BrainACT can be provided to patients aged 18 years or older who are treated by psychologists in hospitals, rehabilitation facilities and mental health care institutions. Patients with an objectified stroke or traumatic brain injury and a score of eight or higher on the depression and/or anxiety subscale of the Hospital Anxiety and Depression Scale^31^ can be considered for treatment. When using antidepressants, the dosage should preferably remain stable during treatment.

### Materials

At the start of the intervention, participants receive a workbook and a USB stick. The workbook contains exercises, visual representations of the metaphors and summaries of each session. The workbook includes two calendars for therapy appointments and the planning of homework assignments. At the beginning of every session, the patient receives the content of that specific session, which is added to the workbook. Furthermore, the USB stick contains the recorded summaries of every session and the mindfulness exercises. A booklet for relatives, containing information on the treatment is included in the workbook. The therapists receive a manual with instructions per session, which also includes the content of the patient's workbook.

### Who Provided: Therapists

The therapy is provided by psychologists with at least two years of experience in acceptance and commitment therapy and working with patients with acquired brain injury. Furthermore, they have completed an acceptance and commitment therapy course of at least five days. In our study, prior to working with the protocol, each therapist received a two-hour training session from the author (JR) to familiarise them with the protocol.

### Delivery: How Much

The BrainACT treatment is developed for individual face-to-face delivery. In total, eight sessions of one hour are included in the protocol. The session titles are ‘Values’, ‘Actions and Mindfulness’, ‘Effect of Control’, ‘Acceptance’, ‘Defusion’, ‘The Self’, ‘Defusion and Mindfulness’ and ‘Resilience’. The sessions are given during a period of three to four months. The first four sessions are given weekly; hereafter, the sessions are given biweekly and between sessions seven and eight there is a break of three weeks.

### Tailoring

The psychologists are free to decide (together with the patient) the order of the sessions, based on information from the intake. The session ‘Values’ can be a helpful start of the intervention when a patient struggles to adjust to the consequences of the acquired brain injury and finds it difficult to shape their post-injury life. However, when a patient is struggling and fighting against negative feelings and thoughts, there might not yet be room for value exploration. In that case, it is advised to start with the session ‘Effect of Control’ followed by the session ‘Acceptance’. Furthermore, it is advised not to postpone the session ‘Values’ until the end of the intervention because the monitoring of committed actions would not be possible with the ‘valuable activity of the week’ exercise. Finally, in several sessions, therapists and patients can choose from different exercises or metaphors, based on what would suit the situation of the patient best.

### Content

The content of the intervention is displayed in Table 2 and illustrated with a case in the accompanying Boxes 1 to 5. The introduction to the case is displayed in Box 1. All sessions generally have the same structure. A session starts with a review of the previous session and the homework exercises. This is followed by an explanation of the acceptance and commitment therapy skill discussed in the particular session using a metaphor. Thereafter the patient and therapist practise this skill together. The session ends with a discussion of the homework for the following week and with a summary of the session. Mindfulness exercises are used as an opening of the session or as a break. The homework exercises always include reading or listening to the summary of the session and practising the acceptance and commitment therapy skill at home.

Box 1 Case introduction, where to go from here

Emma is a 57-year old woman, with a history of endocarditis and heart valve disease, perceptive hearing loss and a burnout, who was admitted to neurorehabilitation by her occupational physician. A year ago, Emma had a right parieto-temporal infarction to the brain, which was initially not recognised as such, but was subsequently confirmed by a magnetic resonance imaging scan. Emma did not succeed in carrying out her administrative job because of difficulty concentrating, fatigue, and experiencing sensory overload. A neuropsychological assessment was performed which showed impairments in visual and verbal mental speed, visual spatial abilities, and mental fatigue. Because of her cognitive and hearing impairments, social interactions were very demanding. Emma had few social contacts and little social support. She did not have the opportunity yet to participate in rehabilitation, had lost her job in the meantime, and felt misunderstood. She did not know where to go from here. She struggled, feeling overwhelmed by emotions and ruminations while resting. Furthermore, she often felt overwhelmed by sensory stimulation and fatigue, while trying to find distraction from these emotions. At the start of the treatment, anxiety was moderate (Hospital Anxiety and Depression Scale score was 14) and depression was mild (Hospital Anxiety and Depression Scale score was 10).

**Table 2.** Overview of the BrainACT intervention

Session title	Content	(Experiential) exercises and metaphors during the session	Homework exercises
Values	Value exploration and defining core values.	- ‘Bus of life’ metaphor - Value sorting exercise - Describing your gravestone or 80th birthday exercise - Writing down core values	- Reading or listening to the summary of this session - Explore which values deserver more or less attention - Valuable pictures exercise
Action and Mindfulness	Committed action in the long and short term in relation to values. Education about mindfulness, and practising contact with the present moment.	- What's the next stop of your bus? (defining short term goals) - Keep driving (defining long-term goals) - Introduction mindfulness: raisin exercise	- Reading or listening to the summary of this session - Perform a daily activity with full attention - ‘Valuable activity of the week’ performing one concrete action that fits within one of the patient's values - Defining obstacles while making these homework exercises
Effect of control	Creative hopelessness; the undeniability of human suffering and the consequences in the long term of trying to control it. Defining strategies used to cope with negative thoughts and emotions.	- Mindfulness exercise: mindful breathing - Identifying how the patient copes with unpleasant experiences. - Tug-of war with a Monster or bouncing ball metaphor	- Reading or listening to the summary of this session - Keeping track of unpleasant thoughts or feelings and how they coped with them - Reread the metaphor of the monster or bouncing ball
Acceptance	Introducing acceptance as an alternative to control.	- Tug-of war with a monster, unwanted guest in your ‘bus of life’, or the finger trap metaphor - Explanation on the difference between pain and suffering with the glass of water exercise - Mindfulness exercise: making space and allowing what is there	- Reading or listening to the summary of this session - Willingness exercise
Defusion	Changing the relationship with thoughts, naming the mind.	- Mindfulness exercise: Attention for thoughts - The passengers in your ‘bus of life’ (post-its) - Naming the mind - Singing difficult thoughts - Mindfulness exercise: floating leaves on a river	- Reading or listening to the summary of this session. - Watch the YouTube video of the ‘bus of life’ - Practice defusion exercises
The Self	Changing the relationship with thoughts about oneself and introducing the constant self.	- Negative and positive labels of oneself - Explanation on the constant self - Mindfulness exercise: mindful movement - Suits (thoughts about oneself) that don’t fit metaphor	- Reading or listening to the summary of this session - Take off a suit that doesn’t fit (anymore) - Practice a mindfulness exercise
Defusion and Mindfulness	Repetition on defusion and mindfulness.	- Mindfulness exercise: the body scan - Defusion exercises: physicalising the thought - Mindfulness exercise: mindful breathing	- Reading or listening to the summary of this session - Practice a mindfulness exercise daily
Resilience	Review of the different core components, explanation on how these skills together lead to psychological flexibility, and preparation on relapse and setbacks.	- Car metaphor - ‘Bus of life’ metaphor and exercise - Strategies on how to keep using acquired acceptance and commitment therapy skills in daily life - Mindfulness exercise: mindful listening	- Reading or listening to the summary of this session - Try to keep practising with the different acceptance and commitment therapy skills - Start integrating acceptance and commitment therapy into your daily life - Live your life as it is valuable to you! Stop fighting, start living!

*Introductory part*. This part is added to the first session to create a safe therapeutic atmosphere and to provide an introduction to and explanation of acceptance and commitment therapy. It is for instance explained that homework is an essential part of the therapy utilising the metaphor of refuelling the car during therapy. Patients come to the therapy to fuel the car but on the other days of the week, patients need to drive themselves. Hereafter, questions such as ‘What do you miss in your life?’, ‘What would you like to do if anything was possible?’, What stands in your way?’, and ‘What stops you?’ are used as an opening of the therapy.

*Session ‘Values’*. Value exploration and clarification are an important part of acceptance and commitment therapy. During this process, the patient might be asked to identify values (what really matters in life) within different life domains such as family, health and spirituality.^19^ Limitations as a consequence of the acquired brain injury might prevent returning to the pre-injury lifestyle and activities. An inquiry into values may help the patient to find new ways of living.

To initiate the value exploration in the BrainACT protocol, the ‘bus of life’ metaphor is introduced.^32^ This metaphor explains that values are the compass that determines the direction of the bus as illustrated in the case description in Box 2. The metaphor is altered to include the consequences of a brain injury. Due to the consequences of the brain injury, the bus might be slower or the road might be bumpier, but the direction of the bus (the values) stays the same.^33^ This metaphor will reoccur in different sessions and is used to illustrate different core components. This session furthermore included the value sorting task. This is a tangible exercise which can provide structure to the value exploration process, which also helped Emma to identify her values as can be read in Box 2. In order to further explore and identify values, the tombstone or 80^th^ birthday^20^ exercises are included.

Box 2 Values and Actions

While introduced to the bus metaphor, Emma realised she felt stuck in her bus, going nowhere. Due to cognitive inability and sadness, she could not think of important values in her life or of a new direction for her bus, by herself. Giving structure to her thinking, with help of the value-sorting task, she could surprisingly easy sort important and unimportant values. The four most important values for her values compass were love, pleasure, contributing and balance.

Emma understood that her feelings of sadness were related to the losses she experienced and had not being able to pursue these important values since she had had the stroke. Committed actions based on her values were engaging in pleasurable activities with loved ones, engaging in satisfying activities by herself, balancing these activities because of fatigue and cognitive impairments and examining if she would like to engage in volunteer work in the future.

Subsequently, Emma engaged more in activities with others. She practiced with undertaking less demanding activities and explaining to others beforehand why she needed to do so. She decided not to engage in volunteer work yet, but instead planned a camper trip with her sister ‘to sort things out emotionally’. She also decided to find a social worker, realising that she needed practical help and support.

At the end of the session, patients can write their most important values in a values compass, which can function as a visual reminder of the patients’ most important values. Besides the summary, the homework exercises include thinking about these most important values and patients are asked to think about values that they gave too much or too little attention, which forms an opening for the session on committed action. In addition, patients are asked to take pictures of valuable moments during the week, as a further value exploration in daily life.

*Session ‘Actions and Mindfulness’*. The next step is to commit to these values set in the value exploration during the session ‘Values’. The ‘bus of life’ metaphor is used again, with bus stops along the way representing different long and short-term goals. These are formulated during the session. Furthermore, obstacles that could occur on the road are discussed. For instance, as described in Box 2, while committing valued actions Emma must take into account fatigue and cognitive impairments.

In the second part of this session, the concept of mindfulness is introduced using the ‘mindfully eating a raisin’ exercise.^34^ Homework exercises include discussing values with someone close and performing a daily activity mindfully. Following this session, the ‘valuable activity of the week’ is added to the homework exercises of every session to encourage behavioural change. This entails performing one specific action that fits within one of the patient's values.

*Session ‘Effect of Control’*. This session starts with the mindfulness exercise ‘mindful breathing’. Hereafter, the therapist and patient identify control strategies the patient uses to control or avoid negative thoughts or feelings. It is explained that these control strategies (for instance avoiding certain situations or drinking alcohol when feeling sad) are often effective in the short term, but are counterproductive in the long term. To experience the effect of control, ‘the tug of war with a monster’ metaphor is introduced and acted out, therapist and patient actively engage in a tug of war.^18^

It is important that next to talking about acceptance and commitment therapy processes patients can also experience them in the therapy room. In Box 3 it is described that Emma realised during the acting out of this metaphor that she will never win this fight.

Box 3 Effect of control and Acceptance

Emma experienced a lot of struggle in her life. She was invited to pull a rope, experiencing the metaphor of the monster. Emma understood ‘I will never win this fight’ but continued to find it difficult to let go of the rope. Within the sessions, when Emma was opening up to feelings and thoughts. She discovered she was in mourning. She could open up to these feelings during the sessions. At home, she was struggling with feelings of grief and sadness, opening up to them from time to time, but wishing them to go away at the same time. At the end of the treatment, she decided to enroll in a therapeutic group with brain injury patients, seeking support in her grieving.

Another option to illustrate control strategies is the ‘the bouncing ball’ metaphor (where negative emotions and thoughts are depicted as a bouncing ball, after which the ball is thrown towards a wall and thus bounces back). The alternative to control is not presented yet to elicit creative hopelessness. Homework includes the daily experiences diary^32^ and rereading the metaphor about the monster or bouncing ball.

*Session ‘Acceptance’*. Acceptance entails tolerating negative and positive thoughts and feelings related to events or circumstances that cannot be changed or one has no control over.^22^ Acceptance, as the alternative to control, is presented in this session with the help of ‘the tug of war with a monster’ metaphor, or ‘the unwanted guest in your bus of life’ metaphor (where the unwanted guest keeps entering your life bus), or ‘the Chinese finger trap’ metaphor.^32^ Therapists make sure that the metaphors are translated to the situation of the participant in the therapy session. For instance, when acting out the tug of war with a monster, it is discussed what the monster of the patient is, how they are fighting with that monster, and what letting go of the rope would mean for the patient. This can also be written down in the working book. These elements are clinically illustrated in Box 3.

The difference between pain and suffering is further experienced in an exercise where patients need to hold a glass of water (unwanted thoughts, feelings, emotions) far away from them (avoidance and control) or on their chest (acceptance). This exercise forms an opening for an explanation on how the resistance or avoidance of pain leads to suffering. The session ends with a mindfulness exercise to practise making space and allowing negative sensations to be present. Homework consists of performing a willingness exercise.^29^

*Session ‘Defusion’*. Instead of altering the content, form, or frequency of thoughts acceptance and commitment therapy focuses on the function of thoughts. Patients are asked to perceive their thoughts more mindfully and to detach from these thoughts.^18^

To practise this, the session starts with the mindfulness exercise ‘attention for thoughts’, where patients observe their thoughts. Hereafter, patients are asked to write their thoughts on post-its and stick them on a picture of the ‘bus of life’, to illustrate the constant stream of thoughts their minds produce. Then, the passengers in the ‘bus of life’ are introduced. These represent the thoughts that keep you from moving in a valued direction. Patients can write the things their passengers tell them frequently around the bus. The session furthermore includes defusion exercises ‘naming the mind’^29^ and an optional exercise of singing a negative thought. The session ends with the exercise ‘floating leaves on a moving stream’.^35^

This session provides therapists and patients with many different forms to practice defusion, some exercises might speak to a particular patient and others might not. For Emma, for instance, the defusion mindfulness exercises helped her to defuse from her thoughts, as described in Box 4. Homework includes watching a movie of the ‘bus of life’ metaphor and practising defusion exercises at home.

Box 4 Defusion and The Self

During meditation exercises such as ‘floating leaves on a moving stream’, Emma could allow thoughts to be present for a while and started to learn to defuse from them. She wrote hindering thoughts around her bus, mostly thoughts related to failure and inability. The exercise ‘suits that don’t fit anymore’ gave her insight in her tendency to avoid conflicts. She started expressing her needs more to others, which increased stress, but also made her more powerful in pursuing her goals.

*Session ‘The Self’*. According to relational frame theory, there are three different senses of self. The conceptualised self (self-as-content) is the image, thoughts, and social roles humans relate to and form of themselves. The experiential self (self-as-process) is the verbal awareness of ongoing psychological experiences and the ability to describe thoughts, feelings and senses in light of the external context. The observing self (self-as-context) is a sense of perspective and a point of view that is unique. The observing self is stable and not influenced by psychological experiences or stressful life events.^18^

To illustrate that everyone has certain ideas and thoughts about themselves, patients write down positive and negative labels about themselves. Hereafter the conceptualised self and the observing self are explained. It is explained that the labels of the previous exercise are part of our self-image (conceptualised self). Another layer of the self (observing self) stays constant despite stressful events such as acquired brain injury. It is furthermore explained that that is the part of the self with which patients can observe the changes following the acquired brain injury.

The experiential self is not included in order to simplify the explanation but is addressed in other ways, through for example mindfulness and defusion exercises. The exercise ‘mindful movements’ is incorporated as a break. Patients perform small movements (for instance lifting the arms) in synchronisation with their breath.

This session ends with the metaphor about suits (thoughts and images about oneself) that don’t fit (anymore).^29^ This metaphor can illustrate the crisis in the conceptualised self which can arise following acquired brain injury.^36^ It is discussed which suits might have fitted well before the injury (the patients might not even have noticed that they were wearing that suit), but now no longer fit. What would it mean if the patient would take off that suit? Box 4 illustrates these exercises in the case description. Homework included trying to take off a suit that does not fit and practising mindfulness exercises.

*Session Defusion and Mindfulness*. This session contains repetition on the core components defusion and mindfulness. It starts with a shortened version of the mindfulness exercise ‘body scan’. Followed by a defusion exercise ‘physicalizing the thought’, where patients are asked to imagine the colour, texture, form etc. of a confronting thought.^37^ The session ends with a mindfulness exercise ‘mindful breathing’. When either the therapist or the patient feels that there is a previous theme or exercise that needs repetition there is room in this session to do so. Homework includes doing a mindfulness exercise daily. These elements in our case description can be found in Box 5.

*Session Resilience*. This session likewise contains repetitions of previous core components and wraps them together. It is explained that the different core components interact and together lead to more psychological flexibility (in the protocol the term resilience is used for simplification).

The ‘bus of life’ metaphor is now discussed and acted out incorporating all the different ACT processes. Just like a normal bus needs all the different individual parts to be able to drive, the bus of life needs all the different core components. The patient drives with all their passengers (obstacles, negative thoughts and feelings) on board, while noticing them but not letting them decide on the route while driving in a valuable direction (an object that presents a value is placed before the patient).

Along the way, patients pass different bus stops representing committed actions. Patients are asked if they can mindfully experience the journey, noticing the sun or a song on the radio. Patients are furthermore asked to look from a perspective at their bus, what would their bus look like if it drove past? Patients might experience how all the different components of acceptance and commitment therapy come together. As described in Box 5, Emma realised that her bus can move in a valued direction with her feelings and thoughts of grief on the passenger seat. Furthermore, strategies on how to keep using acquired acceptance and commitment therapy skills in daily life are discussed. The session ends with a mindfulness exercise ‘mindful listening’.

Box 5 Cognitive Defusion, Mindfulness and Resilience

During the defusing exercise ‘physicalizing the thought’. Emma visualised her grief as a heavy black stone she was carrying. She had the tendency to throw the stone away, but realised that she could not and had to find a way to carry it. Emma learned she could not get rid of difficult feelings such as sadness, grief or insecurity, but could move towards important values taking these thoughts and feelings with her on her bus. In the last session Emma was back from the camper trip with her sister. The trip had given her a lot of experiences. She had experienced that living in the camper, as a different surrounding, had been cognitively demanding, which gave negative feelings and thoughts. Being with her sister had been the most valuable part of the trip. In evaluating the acceptance and commitment therapy with the Bus of Life metaphor, Emma had learned ‘not to stay still, but to keep on driving’, furthermore she realised she needed to accept help from others in order to find the right direction and to pursue future goals related to her values.

## Discussion

Anxiety or depressive symptoms following acquired brain injury are common and there is a need for effective evidence-based treatment options. The potential usefulness of acceptance and commitment therapy for this patient group has been mentioned before^9,10^ and there is growing evidence for its effectiveness for people with acquired brain injury.^12,13,16,37^ Given the need for treatment options for people with mood symptoms following acquired brain injury and the promising results of acceptance and commitment therapy for these patients, the BrainACT treatment was developed.

Strong points of the BrainACT treatment are (i) its adaption to the possible cognitive deficits of patients with acquired brain injury based on previous recommendations,^9,10,26,27^ (ii) the incorporation of the opinions of an expert group, and (iii) feedback from participants and therapists in a pilot study. As a result, we included several recommendations made by O'Cathain, Croot,^38^ such as involving stakeholders, reviewing the research evidence, and designing and refining the intervention during the development process.

General alterations are the use of visual materials, summaries and repetition. Acceptance and commitment therapy specific adaptions include the Bus of Life metaphor as a recurrent exercise, shorter mindfulness exercises, and simplified explanations. Furthermore, the protocol places a strong emphasis on values and monitoring of committed actions as value-driven behaviour has already been found beneficial for people with acquired brain injury.^39,40^ Additionally, the BrainACT intervention includes various experiential exercises and behavioural activation components which are likely helpful therapeutic techniques when providing psychotherapy to people with acquired brain injury.

The BrainACT treatment is an acceptance and commitment therapy covering all the processes included in the psychological flexibility model. The BrainACT treatment is in its essence acceptance and commitment therapy, no other theories or approaches, such as psycho-educations on the consequences of acquired brain injury, were integrated into the protocol; the goal was to examine whether acceptance and commitment therapy can contribute to well-being following acquired brain injury. When this approach is found beneficial it could be integrated into a holistic neurorehabilitation context and/or combined with psycho-education.

Theoretically, the six core components of acceptance and commitment therapy can contribute to the adaptation process following acquired brain injury.^9,10^ Whether these components individually contribute to the enhancement of psychological flexibility should be further investigated. The psychological flexibility model has been validated in people with chronic pain,^41^ but this has not yet been done for people with acquired brain injury.

Some cautionary remarks should be made. First, while adjustments were made for possible deficits following acquired brain injury, it is still possible that the intervention is too demanding for some patients, for instance, patients with aphasia.

Next, as is common in acceptance and commitment therapy, metaphors were utilised to illustrate its different core components. Metaphors have been proven useful in other therapies for people with acquired brain injury, such as metaphoric identity mapping.^42^ Nevertheless, previous studies have shown that people with acquired brain injury can have difficulties with figurative speech.^43^ That study was, however, not performed within a therapy setting. Hains^44^ showed that patients with schizophrenia can understand metaphors used in therapy better than metaphors used in a test environment and the use of metaphors in our pilot study was feasible.

Furthermore, we did not define nor explore the optimal timing for the BrainACT intervention following the acquired brain injury. The psychological adaptation process following acquired brain injury can be a long road, encompassing several years. It is not clear when the BrainACT intervention would be most helpful in this process.

Lastly, as with most interventions, this is not a ‘one size fits all’ approach. For some patients, different psychotherapies might be more appropriate.

The next step will be to examine the feasibility, clinical effectiveness, and cost-effectiveness of this intervention. This is currently being investigated in a multicentre randomised controlled trial with a one-year follow-up period^17^ in which the BrainACT intervention is compared to a combined psycho-education and relaxation treatment.

**Clinical messages**

- Acceptance and commitment therapy is a promising intervention for treating people with acquired brain injury who experience anxiety and depressive symptoms.
- The BrainACT treatment is an adapted acceptance and commitment therapy intervention for people with acquired brain injury and possible cognitive deficits.
- The BrainACT treatment is currently being evaluated in a randomised controlled trial.
